# Predicting age and mass at maturity from feeding behavior and diet in *Manduca sexta*: An empirical test of a life history model

**DOI:** 10.1002/ece3.9848

**Published:** 2023-02-22

**Authors:** Anna L. Parker, Anna Albright, Joel G. Kingsolver, Geoffrey Legault

**Affiliations:** ^1^ University of North Carolina – Chapel Hill Chapel Hill North Carolina USA

**Keywords:** body size, diet quality, feeding behavior, life history, stochastic modeling

## Abstract

Feeding for most animals involves bouts of active ingestion alternating with bouts of no ingestion. In insects, the temporal patterning of bouts varies widely with resource quality and is known to affect growth, development time, and fitness. However, the precise impacts of resource quality and feeding behavior on insect life history traits are poorly understood. To explore and better understand the connections between feeding behavior, resource quality, and insect life history traits, we combined laboratory experiments with a recently proposed mechanistic model of insect growth and development for a larval herbivore, *Manduca sexta*. We ran feeding trials for 4th and 5th instar larvae across different diet types (two hostplants and artificial diet) and used these data to parameterize a joint model of age and mass at maturity that incorporates both insect feeding behavior and hormonal activity. We found that the estimated durations of both feeding and nonfeeding bouts were significantly shorter on low‐quality than on high‐quality diets. We then explored how well the fitted model predicted historical out‐of‐sample data on age and mass of *M. sexta*. We found that the model accurately described qualitative outcomes for the out‐of‐sample data, notably that a low‐quality diet results in reduced mass and later age at maturity compared with high‐quality diets. Our results clearly demonstrate the importance of diet quality on multiple components of insect feeding behavior (feeding and nonfeeding) and partially validate a joint model of insect life history. We discuss the implications of these findings with respect to insect herbivory and discuss ways in which our model could be improved or extended to other systems.

## Graphical abstract

Animal life history (e.g., age and mass at maturity) is the outcome of complex interactions between feeding behavior, diet quality, and developmental traits. We examined these connections by combining data from laboratory feeding trials with a joint model of insect growth and development for a larval herbivore, *Manduca sexta*. The model fit the data well and provided a formal link between different diets, their effects on feeding behavior, and the resulting differences in age and mass at maturity, illustrating what insights are possible when multiple components of feeding behavior and development are jointly modeled.

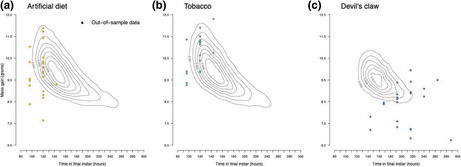

## 1 INTRODUCTION

All animals eat, but they eat in many different ways. Many predators consume large prey in a single meal, with long time periods of nonfeeding between meals. By contrast, some grazers eat continuously for extended periods of time, spending a large fraction of their time actively consuming food. Most insects and other herbivores have distinct feeding “bouts” (i.e., periods of active consumption) alternating with nonfeeding bouts (Karasov & Martinez del Rio, 2020). This delineation divides feeding behavior into two analyzable components (Nielsen, 1999), each of which is known to affect physiological processes such as digestion and assimilation (Harrison et al., 2012; Woods & Kingsolver, 1999), growth and development (Anderson & Cummins, 1979; Shobana et al., 2010), and immune response (Lee et al., 2008). These processes are known to contribute to the expression of life history traits, including age and size at maturity, mating success, and fecundity, which together determine lifetime fitness (Harari et al., 1999; Stoffolano et al., 2000).

There is a well‐established empirical and theoretical literature that links foraging decisions among alternative food resources to life history and fitness (Boggs, 1992; Lee et al., 2004; Perry & Pianka, 1997; Raubenheimer & Simpson, 2018). For example, foraging theory explores how time spent in a resource patch and the transit time between patches varies with resource quantity and quality, and how foraging decisions determine mean fitness (Boggs, 1992; Stephens & Krebs, 1986). By contrast, few studies have explicitly linked how resource‐related changes in feeding behavior during foraging (e.g., feeding bout length) affect growth, development, and fitness (Shelton & Mangel, 2012; Simpson & Abisgold, 1985). What determines the duration of feeding and nonfeeding bouts, and how do these vary with resource quality and through ontogeny? Do individuals in a population vary in their feeding characteristics? How do decisions about when to eat and not to eat determine body size, development time, and fitness? These questions highlight the need for a theoretical and empirical framework for understanding the causes and consequences of variation in feeding behaviors, especially in organisms where foraging choices are limited (Karasov & Martinez del Rio, 2020; Penry & Jumars, 2008).

Modifications to feeding behavior in response to diet quality are frequent and well‐documented in many organisms (Colasurdo et al., 2007; Johnson & Lincoln, 1991; Lincoln et al., 1986; Lincoln & Couvet, 1989; Raubenheimer & Simpson, 2018) and can have strong effects on life history traits. For example, in the absence of behavioral or physiological adjustments, individuals consuming different quality diets will diverge in development time, size at maturity, and fecundity (Awmack & Leather, 2002; Simpson & Raubenheimer, 1993), though different traits may be optimized under different nutritional proportions (Rapkin et al., 2018). When considering insect herbivores and their hostplants, diet quality is determined by both the available nutrients and defensive compounds present (Couture et al., 2016; Hägele & Rowell‐Rahier, 1999; Lincoln & Couvet, 1989; Raubenheimer & Simpson, 2003). Some insect herbivores will modify their foraging behavior based on the quality of the resource, either by selecting a different resource (Thorsteinson, 1959) or by selecting a combination of different available resources (Ali, 2022; Waldbauer & Friedman, 1991). For example, many Orthopterans given a choice of several different resources will feed selectively in order to maintain a consistent ratio of macronutrients in their diet (Clissold et al., 2014). This is facilitated by the dispersal capabilities of Orthopteran nymphs and adults (Bazazi et al., 2012; Simpson & Sword, 2008).

For many larval insect herbivores, however, choosing alternative resources to feed on is not possible: an individual larva may remain and feed on a single hostplant individual for its entire larval life. As a consequence, the behavioral responses of a larva to a food resource may be largely limited to changes in the duration of feeding and nonfeeding bouts and in the proportion of time spent feeding. When restricted to a single, lower‐quality food resource, insect herbivores frequently increase their consumption rate, partially (or fully) compensating for the reduced resource quality (Couture et al., 2016; Slansky & Wheeler, 1992). While studies have investigated the effects of compensatory feeding on life history traits (Couture et al., 2016; Cruz‐Rivera & Hay, 2000; Slansky & Wheeler, 1992), they rarely link increased rates or amounts of food consumption to changes in the length or number of feeding bouts. Two notable exceptions are Reynolds et al. (1986), which found that the duration of feeding bouts changed with diet in the tobacco hornworm, *Manduca sexta*, and Timmins et al. (1988), which investigated how diet type affected bout duration and conversion rate. However, neither study attempted to link these changes with development time or other life history traits. In order to mechanistically model how feeding relates to growth and development, this connection must be made.

Recently, Legault and Kingsolver (2020) proposed an insect life history model that incorporates feeding behavior and hormonal changes throughout larval development to predict the joint distribution of age and mass at maturity, two life history traits with strong fitness consequences (Nijhout et al., 2006). At its core, the model assumes that developing larvae continuously alternate between feeding and nonfeeding states, gaining mass only while feeding. Mass gained while feeding is assumed to be directly proportional to larval feeding time. The model is based on the well‐known developmental biology of *Manduca sexta* larvae (Nijhout et al., 2006); however, it has yet to be validated with empirical data on insect feeding behaviors, including across different resources, and it is unknown how well its predictions align with observed life history traits in insects.

We combined experimental data with this mechanistic life history model to address two questions about the connections between feeding behavior, food quality, and life history traits: (1) How does food quality affect the temporal patterns of feeding behavior; and (2) How do differences in feeding behavior affect life history traits? We conducted observations of individual *M. sexta* 4th and 5th (final) instar larvae feeding on three diet types, including two hostplants and an artificial diet, each of differing qualities. We then used these data to parameterize the life history model, estimating key model parameters including the total time spent feeding during a trial for the different diet types. Finally, we used the fitted model to predict the ages and masses of larvae at the end of 4th and 5th instars and compared these predictions to independent out‐of‐sample data. Our results support the validity of this model as a predictive tool, especially for feeding during the 4th instar, and also highlight how differences in feeding behavior between individuals and across diet types have strong effects on growth rate, final body size, and development time.

## 2 METHODS

### 2.1 Study system

For *M. sexta*, plants from the family Solanaceae such as tobacco (*Nicotiana tabacum*) and jimsonweed (*Datura* sp.) serve as its usual hostplants (Madden & Chamberlin, 1945). However, in the southwestern part of *M. sexta*'s range, devil's claw (*Proboscidea* sp.) has been found to be an alternate host (Mira & Bernays, 2002). Recently, the devil's claw species *Proboscidea louisianica* has invaded the southeastern United States (Correll & Johnston, 1970), allowing for its use as a hostplant by *M. sexta* in the region. Devil's claw is a low‐quality host: caterpillars reared on devil's claw leaves have higher mortality, smaller masses at maturity, and longer development times than those raised on tobacco or standard artificial diet (Diamond et al., 2010; Diamond & Kingsolver, 2010a, 2010b).

We collected *M. sexta* eggs from a colony population at the University of North Carolina, Chapel Hill, for this experiment. This colony of *M. sexta* has been domesticated for approximately 250 generations (Kingsolver, 2007), during which time they have been fed artificial diet and maintained at 25–26°C constant temperature, with a 14L:10D light cycle in a large environmental chamber. No wild‐caught individuals have been added since domestication. The eggs collected for this experiment were hatched and maintained under standard larval rearing conditions (25°C constant temperature, 14L:10D light cycle) in a Percival model VL‐36 environmental chamber. From hatching, we fed individuals ad libitum on one diet type throughout development, either standard artificial diet or leaves from devil's claw or tobacco grown in the UNC greenhouse (rearing temperature 26–28°C, 14L:10D light cycle). We tracked larvae individually after hatching and recorded the date and mass at molt to third, fourth, and fifth instar (as indicated by the presence of a shed headcap and the width of the head in relation to the thorax). Our sample sizes differed slightly between the start of the fourth instar (diet *N* = 18, tobacco *N* = 19, devil's claw *N* = 11) and the start of the fifth instar (diet *N* = 17, tobacco *N* = 10, devil's claw *N* = 11), due to differential mortality across diet types. Due to university restrictions for research under the COVID‐19 pandemic, we could not increase our sample sizes to correct for this.

### 2.2 Feeding trials

We observed each individual twice: once on the day after molt to 4th instar and once on the day after molt to 5th instar. One hour before each trial, we massed individuals and placed them in a dish without food, to induce a feeding response. During each trial, we placed individuals on top of their food source and continually observed for 1 h. An individual was considered “feeding” when its mouth was seen moving while in contact with the food source and when plant/diet material was visibly being diminished. We recorded the timing of each switch between states of feeding and nonfeeding, to the nearest second. If the length of a feeding or nonfeeding bout was less than 5 s, that bout was disregarded, and the initial state was maintained.

### 2.3 Analysis of feeding behavior

**FIGURE 1:**
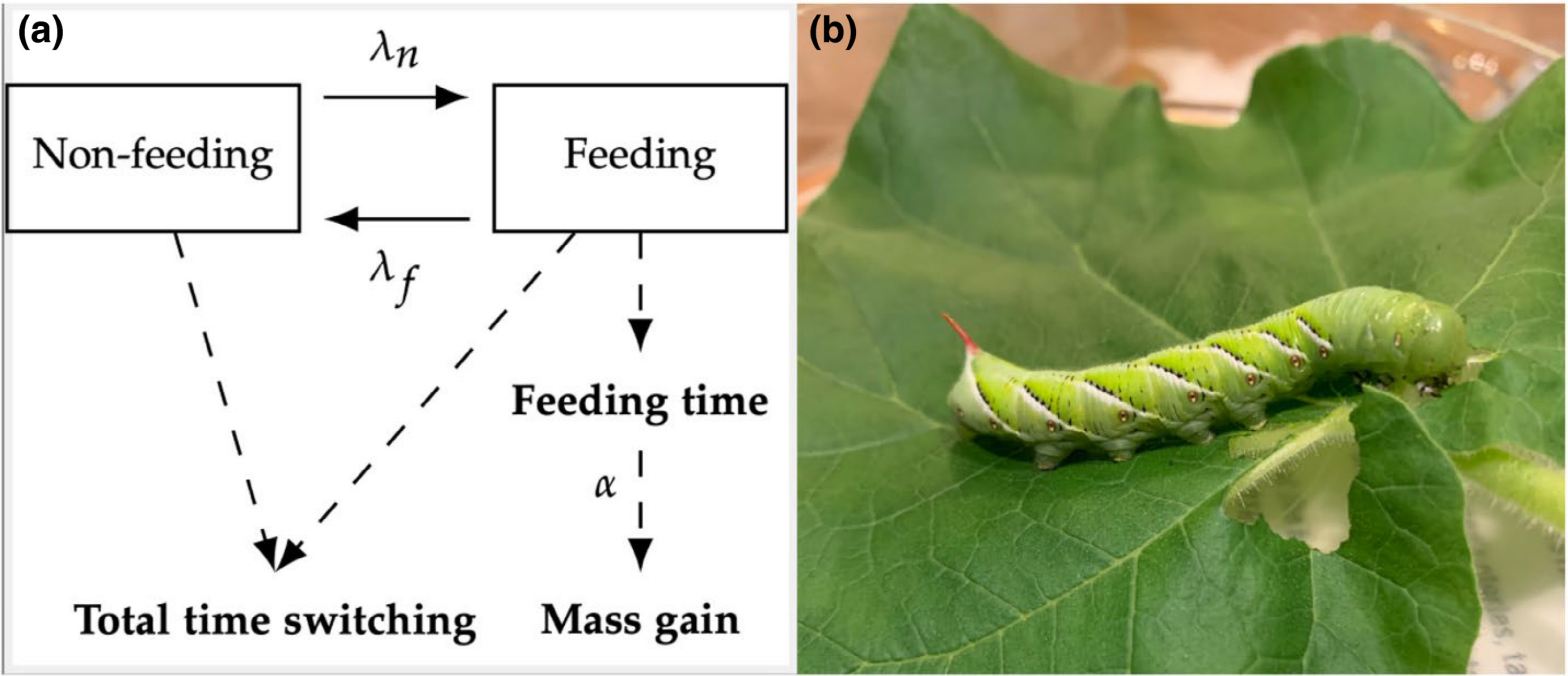
(a) Schematic representing the portion of the model from Legault and Kingsolver (2020) parameterized by our experiment. *λ*_n_ denotes the rate of switching from nonfeeding to feeding, *λ*_f_ represents the rate of switching from feeding to nonfeeding, and *α* denotes the mass gain per unit time spent feeding. Values for the three remaining parameters (not pictured) can be found in the literature (Legault & Kingsolver, 2020). Figure adapted from figure 1a in Legault and Kingsolver (2020). (b) Image of a 5th instar *M. sexta* larva consuming a *P. louisianica* leaf during a feeding trial. The mandibles are clearly visible, allowing for recording of switches between feeding and nonfeeding bouts.

We focused on parameterizing the submodel for mass gain from Legault and Kingsolver (2020), which simplifies feeding behavior by assuming that individual caterpillars continuously switch between two states: (1) nonfeeding, where they gain no mass, and (2) feeding, where they gain mass. For this submodel, the waiting times between switches (i.e., the duration of individual feeding/nonfeeding bouts) are assumed to be exponentially distributed, and each state can have a different switch rate, *λ*_f_ for switching out of feeding and *λ*_n_ for switching out of nonfeeding (Figure 1). Values of *λ*_f_ and *λ*_n_ reflect the average number of switches per unit time, with higher values reflecting faster switching. Thus, if a caterpillar spends long periods of time masticating plant material, the feeding switch rate *λ*_f_ would be low, and likewise if they spend very brief periods digesting food, *λ*_f_ would be high. Another parameter, *α*, represents the amount of mass gained per unit time spent feeding and relates to the ability of the individual to process the food resource and absorb nutrients (Figure 1). Mass gain can therefore be calculated by multiplying α against the total time spent feeding. We note that *α* is determined by multiple processes, including the bite size and biting rate during feeding, digestive efficiency and metabolic rates, that may vary with food resource, temperature, and other environment factors.

For each of the two instars and three diet types, we estimated parameters *λ*_f_ and *λ*_n_ by forming a likelihood function that takes the product of the probability densities given by $f\left(x|Z,\Theta \right)$, where x is the total time spent feeding by a larva during a feeding trial, Z is the length of the feeding trial (1 h), and Θ is the vector of the parameters *λ*_f_ and *λ*_n_. This probability density function is the same as the mass gain probability function derived for the development model in table 1 of Legault and Kingsolver (2020), except that it is not scaled by a mass gain term, since we did not directly observe mass gain during the feeding trials. To find the vector Θ that maximized the likelihood, we did numerical optimization using the “optim” function in R using the conjugate gradient method.

We used nonparametric bootstrapping (Davison & Hinkley, 1997) to generate 90% confidence intervals, which we use to both quantify uncertainty and compare parameter estimates for different diet types. This was done by optimizing the likelihood function to 100,000 samples (with replacement) of each data set and then finding the 90% percentile bootstrap interval of the estimates. We considered the feeding/nonfeeding rates to differ significantly between diet types if these 90% confidence intervals did not overlap.

For each diet type and instar, we separately estimated the α parameter of the development model, which represents the amount of mass gained per unit time spent feeding. It was calculated using both empirical data and the estimated values of *λ*_f_ and *λ*_n_ (see above). For each individual larva, we used two mass measurements recorded while conducting our feeding trial experiment: mass at molt to 4th or 5th instar and mass 1 h before each trial. These measurements occurred 24 ± 2 h apart, recorded to the quarter hour. These values were then divided by expected value of the proportion of time spent feeding during this 24‐h period, which was based on the estimated *λ* parameters above and the assumption that larvae were switching continuously during this period.

### 2.4 Model validation and prediction

To assess model fit, for each diet type we simulated (*n* = 100,000) feeding trials using the estimates for *λ*_f_ and *λ*_n_. Simulated individuals started in a nonfeeding state and randomly switched between states for the equivalent of an hour (the length of our empirical trials). We then computed the total time spent feeding in each simulated trial, producing samples of the total duration of feeding, which we compared with our empirical observations.

To assess the validity of our fitted model more generally, we also compared model predictions to previously collected, out‐of‐sample data on 4th and 5th instar *M. sexta* raised on similar diets (Diamond & Kingsolver, 2010a; Kingsolver et al., 2009). This out‐of‐sample comparison measures the model's predictive accuracy (Tredennick et al., 2021) and for several reasons should be regarded as a conservative test of model validity. First, out‐of‐sample data are, by definition, data the model was not trained on, and predicting outcomes for novel situations is in general difficult. Second, our out‐of‐sample data on *M. sexta* are 14 years removed from the training data, meaning there could be genetic differences between populations related to development and feeding behavior. Thus, we do not necessarily expect strong overlap between model predictions and out‐of‐sample data; rather, the comparison serves mainly as a check on whether the model is able to describe at least some of the key features of insect development in a novel context.

We first compared model predictions of mass gain during the 4th instar to the out‐of‐sample data on 4th instar mass gain. We simulated the model (*n* = 100,000) using the point estimates of *λ*_f_ and *λ*_n_ for each diet type, tracking the time spent feeding. To convert feeding time to mass gain, we sampled *α* from a normal distribution that had been fit to the individual *α* values calculated above. In order to focus on mass gain specifically, the duration of the 4th instar in these simulations was obtained by sampling from the reported 4th instar durations of the independent study.

To compare model predictions with the out‐of‐sample 5th instar data, we simulated (*n* = 100,000) the full joint model of Legault and Kingsolver (2020), which includes both switching between feeding and nonfeeding states, as well as the degradation of juvenile hormone (JH). As above, for each diet type we used the *λ*_f_ and *λ*_n_ point estimates, and samples from a normal distribution fitted to the individual *α* values. For the simulations, initial masses (i.e., mass at the start of 5th instar) were sampled from a normal distribution fitted to the initial masses of the out‐of‐sample data. The full model requires three additional parameters: critical weight, *w*_c_, the mass where JH begins to degrade (complete degradation triggers the cessation of growth); the amount of JH, *j*; and the degradation rate, *μ*. The critical weight for *M. sexta* is approximately 7 g under similar laboratory conditions (Davidowitz & Nijhout, 2004) and so we set *w*_c_ = 7. The duration of the cessation of growth phase for *M. sexta* at 25°C is approximately 48 h (Davidowitz & Nijhout, 2004), which we assumed was the mean duration of this phase in the full model, leading us to select *j* = 48 and *μ* = 1 (duration is gamma distributed with mean *μj*). After conducting this analysis, we could compare the model predictions to the observed data, thus assessing the model's utility as a predictive tool for *M. sexta* growth and development.

## 3 RESULTS

### 3.1 *λ*_n_ and *λ*_f_

**FIGURE 2:**
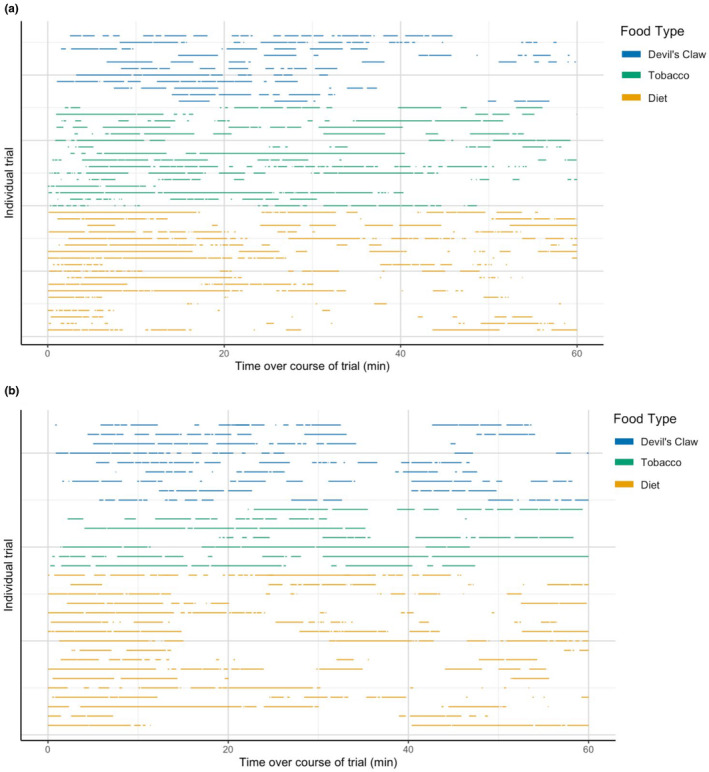
Representation of individual feeding trials across (a) 4th and (b) 5th instars. Each horizontal row represents one feeding trial by an individual feeding on devil's claw (blue), tobacco (green), or diet (yellow). Each segment of colored horizontal line indicates a period of feeding, with the width denoting the length and timing of that period. Blank spaces indicate periods of nonfeeding.

Across diet types, there was high variability in the timing and duration of feeding and nonfeeding bouts (Figure 2). Based on the estimated 90% confidence intervals, for 4th instar larvae, feeding switch rates were significantly higher (i.e., larvae switched out of feeding significantly faster) on the low‐quality resource (devil's claw) compared with the two high‐quality resources (artificial diet and tobacco), both of which had overlapping intervals (Table 1). Similarly, the nonfeeding switch rate on the low‐quality resource (devil's claw) was significantly higher than switching on artificial diet, and only minorly overlapped with the nonfeeding switch rate on tobacco (Table 1). These findings were consistent with the 5th instar results, insofar as the 90% confidence intervals of the switching rates on the low‐quality resource (devil's claw) never overlapped with the intervals for the highest quality resource (artificial diet) and had only moderate overlap with the intervals for tobacco *λ*_n_ (Table 1). The distribution of mass gain while feeding α differed between instars (higher during 5th instar) but did not differ between diet types (Table 2).

**TABLE 1:** Parameter estimates with units of switches per hour across diet types for 4th and 5th instar larvae.

	Artificial diet	Tobacco	Devil's claw
4th instar			
Feeding switch rate	10.25 (7.48, 18.24)	16.21 (11.72, 30.84)	56.22 (36.33, 147.69)
Nonfeeding switch rate	4.93 (3.00, 10.19)	10.19 (7.32, 19.52)	24.36 (16.85, 57.95)
5th instar			
Feeding switch rate	16.85 (12.59, 29.53)	10.7 (6.2, 42.61)	59.43 (40.81, 187.89)
Nonfeeding switch rate	11.05 (8.15, 18.91)	12.63 (8, 46.23)	37.99 (25.23, 128.16)

*Note*: Point estimates are shown, with the bootstrapped 90% confidence intervals of the estimates shown in parentheses.

**TABLE 2:** Estimated mass gain (grams) per hour while feeding, α, across diet types for 4th and 5th instar larvae.

	Artificial diet	Tobacco	Devil's claw
4th instar	0.04 (±0.02)	0.05 (±0.01)	0.04 (±0.01)
5th instar	0.15 (±0.04)	0.11 (±0.03)	0.14 (±0.03)

*Note*: Means and standard deviations (in parentheses) are shown by diet type.

### 3.2 Assessment of model fit

The model assumes the lengths of feeding and nonfeeding bouts are exponentially distributed. We found that the fitted exponential curves aligned well with our data on bout length for larvae fed devil's claw (Figures S1 and S2, panels C and F). The fitted curves also matched our data on bout length for artificial diet and tobacco (Figures S1 and S2, panels A, B, D, E) except that they somewhat underpredicted the number of short feeding bouts on artificial diet and tobacco, possibly due to low sample size and estimates being influenced by a higher‐than‐expected number of lengthy feeding bouts (Figures S1 and S2).

**FIGURE 3:**
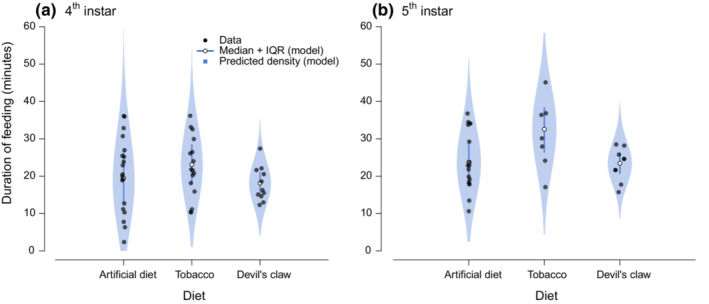
Violin plots demonstrating model fit for 4th (a) and 5th (b) instar larvae. The raw data for total time spent feeding in each trial (black dots) are jittered within diet type. The white dot and thin line represent the median and interquartile range for the model predictions, with the estimated density shown in blue violins.

We also assessed model fit by comparing the observed total times spent feeding during the 1‐h feeding trials to simulations of the fitted model. Median feeding time did not differ substantially between diet types, but there was greater variation in total time spent feeding for larvae on diet and tobacco than for those on devil's claw (Figure 3, black dots). Model predictions correctly capture both these patterns in the data (Figure 3, blue violins).

### 3.3 Assessment of model validity

**FIGURE 4:**
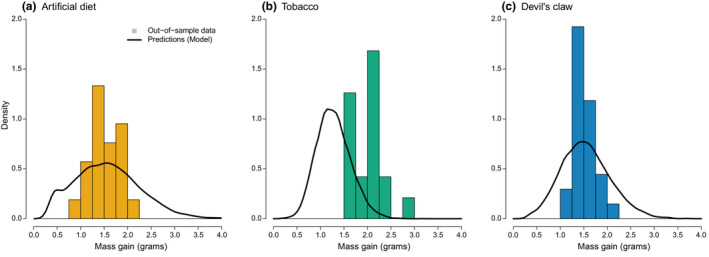
Density plots of total mass gain in grams over the course of the 4th instar for caterpillars feeding on artificial diet (a), tobacco (b), and devil's claw (c). Bars show the densities of independent, out‐of‐sample data (Diamond & Kingsolver, 2010a; Kingsolver et al., 2009), while the solid lines show model predictions.

Finally, we assessed the validity of the model as a general description of feeding behavior and life history in *M. sexta* by examining how well it could predict out‐of‐sample 4th and 5th instar data from two previous studies (Diamond & Kingsolver, 2010a for tobacco and devil's claw, Kingsolver et al., 2009 for artificial diet). First, we estimated the average mass gain during the 4th instar using the fitted model (as described in Methods: *Model validation and prediction*) and compared this to the empirical out‐of‐sample developmental data. The fitted model predicted mass gain for out‐of‐sample larvae fed artificial diet and devil's claw (Figure 4a,c, alignment of histogram mean with prediction mean), but underestimated mass gain for larvae fed tobacco (Figure 4b). In addition, model predictions exhibited greater variation in mass gain than was observed on artificial diet and devil's claw (Figure 4a,c).

**FIGURE 5:**
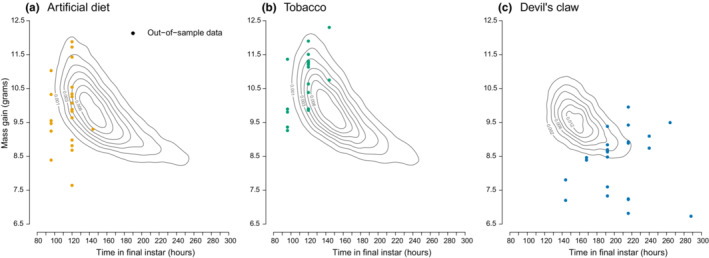
Plots comparing model predictions to out‐of‐sample empirical data, including variation inherent to the model and due to differences in mass gain observed. The labeled contours are estimates of the joint density of masses and ages at maturity (wandering) predicted by the model for individuals raised on artificial diet (a, yellow), tobacco (b, green), and devil's claw (c, blue). The colored points are data on age and mass at wandering of individuals, collected from out‐of‐sample datasets from previously published Kingsolver lab studies (Diamond & Kingsolver, 2010a; Kingsolver et al., 2009).

With respect to the 5th instar life stage, the predicted joint distribution of age and mass at maturity was misaligned with the out‐of‐sample data (Figure 5). In particular, based on the extent of the experimental data and the size of the 90% contours of the predictions, variation in development time was smaller for out‐of‐sample larvae fed artificial diet or tobacco compared with predictions (Figure 5a,b, *x*‐axis), and larger for out‐of‐sample larvae fed devil's claw (Figure 5c, *x*‐axis). The covariance between mass gain and development time—in essence the shape or orientation of the two variables—also differed markedly between the data and the model (Figure 5). However, the marginal distribution of mass gain (Figure 5, *y*‐axis) matched relatively well with observed mass gain for both artificial diet and tobacco (Figure 5a,b).

## 4 DISCUSSION

Understanding the ecological and evolutionary ramifications of diet choice requires linking diet‐dependent feeding behavior to life history traits/outcomes, such as age and mass at maturity. Here, we have connected these processes using a combination of empirical observations and mechanistic modeling. Our results show that *M. sexta* feeding behavior does change with diet type, and this change leads to differences in age and mass at maturity. Below, we discuss the implications of our results and the strengths and limitations of our approach and finally suggest how to adapt the approach to other species and systems.

### 4.1 Diet‐level differences and model predictions

For animals restricted to a single food resource, feeding behavior is usually quantified using a single consumption rate, reflecting the average amount of food consumed during a time interval. Numerous studies have documented that dietary factors can alter mean consumption rate. For example, many insect herbivores exhibit compensatory feeding, in which reduced diet quality increases mean consumption rates (Cruz‐Rivera & Hay, 2000; Simpson & Raubenheimer, 1993; Slansky & Wheeler, 1992). However, these results only tell part of the story, since consumption during an interval is actually determined by the combination of feeding and nonfeeding bouts, each of which may respond differently to diet quality. Simpson and Abisgold (1985) is one of the few studies that consider the mechanisms underlying increased consumption rates, showing that in locusts, reduced dietary protein reduced nonfeeding bout length but not feeding bout length or meal size, thereby increasing consumption rate. By contrast, our results show that *M. sexta* fed the lower‐quality devil's claw exhibited shorter feeding and nonfeeding bouts (and thus faster switching rates) compared with individuals fed higher‐quality diets (Table 1). This behavioral response reduced the proportion of time spent feeding and thus decreased consumption and growth (Figures 3 and 4; Figure S1). In contrast to our results, in which behavior was similar on tobacco and artificial diet, Reynolds et al. (1986) show that, in *M. sexta*, feeding bouts were significantly shorter for individuals fed artificial diet compared with tobacco leaves, resulting in less time spent feeding (though not decreased growth).

During the observational trials, we noticed that the shorter feeding bouts on devil's claw were accompanied by increased movement about the leaf surface compared with the other treatment groups (A. Albright, personal observation), indicative of a searching pattern (Bell, 1990). The caterpillars seemed to be sampling different sections of the leaf surface: moving to a location, feeding for a brief period of time, and then moving on to a different location. This sampling was repeated rapidly, contributing to the shorter feeding and nonfeeding bout lengths on devil's claw. One potential explanation for this is that devil's claw lacks the alkaloid compounds typical of tobacco and other Solanaceous hosts that stimulate feeding behavior in *M. sexta* (de Boer & Hanson, 1987). Similarly, the distinctive defensive secondary compounds found in devil's claw may explain the absence of compensatory feeding of *M. sexta*, which we would expect if leaf nutritional quality were driving feeding behavior (Riffle et al., 1990, 1991).

Based on our model, which fits the data well, diet‐level differences in feeding behavior alone can substantially alter the distribution of life history traits, particularly during the 5th instar (Figure 5). Compared with a high‐quality diet (artificial diet or tobacco), low‐quality diet (devil's claw) leads to faster switching out of nonfeeding and much faster switching out of feeding (i.e., shorter feeding bouts). This in turn leads to longer development time (Figure 5c, *x*‐axis) and lower mass at maturity (Figure 5c, *y*‐axis). In our model, such changes occur independent of changes to feeding efficiency (i.e., conversion of feeding time to mass gain). These predictions partially align with the out‐of‐sample data used to assess model validity. In particular, the fitted model accurately predicts expected mass gain during the 4th and 5th instar for two out of three diet types. Thus, our model provides an accurate and generalizable description of a key aspect of *M. sexta* life history. Moreover, it implies that life history outcomes in *M. sexta* can be strongly influenced solely by stochasticity and diet‐induced behavioral changes.

Predictions from the fitted model were misaligned with 5th instar out‐of‐sample data on development time (Figure 5, *x*‐axes) and mass gain (Figure 5, *y*‐axes), particularly for devil's claw. This differed from the results for the 4th instar, where predicted mass gain closely resembled the out‐of‐sample data (Figure 4). This discrepancy is likely due to a combination of factors, chiefly the biological complexity of the 5th instar. In the model, development time is assumed to be determined by a mass threshold (i.e., critical weight), which triggers the stochastic degradation of a single hormonal control molecule. In reality, there are a series of hormonal changes and controls on development that occur throughout the 5th instar. For instance, as larvae proceed through the 5th instar, the titer of JH decreases and then prothoracicotropic hormone (PTTH) and ecdysteroids are secreted (Nijhout et al., 2006). These additional molecules signal the cessation of feeding and the beginning of wandering (Nijhout et al., 2006). Crucially, PTTH is only secreted during a daily, 8‐h window of time, called the “photoperiodic gate” (Truman, 1972; Truman & Riddiford, 1972), which our model does not take into account. This complexity may cause the difference in estimation accuracy between the 4th and 5th instar analyses. Finally, it is worth noting that there was a 14‐year separation between the training and the out‐of‐sample datasets we considered. While the laboratory colony of *M. sexta* used for both sets of experiments was the same, it had undergone a minimum of 70 generations of nonrandom evolution since the out‐of‐sample data had been collected. The plant strains utilized in the experiments also differed. Thus, the fact that the model accurately recapitulates the out‐of‐sample data on mass gain during the 4th instar data is, in our view, fairly remarkable and speaks to the robustness of the model.

### 4.2 Variation included and excluded from model framework

A major strength of this model is that it combines multiple forms of individual variation. For instance, in addition to variation arising from random switching between feeding and nonfeeding, the model incorporates individual variation in initial mass and mass gain per unit time feeding. While we assume such variation is “random” in the sense that we do not attempt to explain its source, in principle this structure could be used to account for otherwise unobserved biological processes. For example, mass gain per day has been shown to be heritable and differentiated between genetic lines (Cammack et al., 2005), and thus, the distribution of *α* could be seen as a measure of the genetic variation within a population. Similarly, other feeding behaviors are known to have a genetic basis (Cammack et al., 2005; Chinchilla‐Ramírez et al., 2020; Newmyer et al., 2019) and could be represented with parameters from our model.

Other important sources of variation from this experiment, however, would require further extensions. For instance, differences in individual leaf quality within treatment groups may lead to the observed variation in behavior. Variation in nutrient and secondary compound concentrations between leaves of different ages can be quite high and can influence insect herbivore life history traits (Center, 1987; Feeny, 1970; Kause et al., 1999; Nelson et al., 1981). We attempted to minimize interleaf quality differences by collecting similarly aged and sized leaves for each of our feeding trials; however, some differences in leaf quality among replicates may still have been present. Assuming these differences were uncorrelated with diet treatment, they were unlikely to have biased our results. If future studies were interested in investigating more subtle effects of diet quality, such as within‐plant resource heterogeneity, such effects could be incorporated by, for example, allowing the distribution of mass gain per unit time feeding to depend on diet or leaf type. This would likely require additional experiments to find the distributions and parameterizations that best explained mass gain for different plants/leaves.

### 4.3 Future directions

The factors described previously may account for some of the discrepancies between model predictions and our empirical data, suggesting that a more complex model could better explain the link between feeding behavior and life history. But the accuracy of the fitted model during feeding trials and the hormonally‐stable 4th instar shows that even in its present form, it can be a useful framework for understanding the causes and consequences of diet‐induced changes to feeding behavior in *M. sexta*. Further, we believe our model could be adjusted for specific taxa of interest, especially insect taxa. The small number of parameters in our model (λ_f_, λ_n_, *α*, and *w*_c_) lessens the amount of data required for training, increasing the ease of customization to other systems.

In a world increasingly affected by anthropogenic climate change, predicting how species' behavior changes with different circumstances is vital, because patterns of herbivore consumption are likely to change with increasing temperatures (Clissold & Simpson, 2015; Coggan et al., 2011; Lee et al., 2015; Lemoine et al., 2013) and atmospheric carbon dioxide levels (Zavala et al., 2013). In addition, recent theoretical and empirical studies illustrate how increasing temperatures and reduced resource quality or quantity can interact to negatively impact optimal temperatures, growth rates, and fitness (Clissold & Simpson, 2015; Huey & Kingsolver, 2019; Thomas et al., 2017). An integrative approach that accounts for individual‐ and diet‐level variation in herbivore outcomes will be critical to ensuring the robustness of such predictions. Future empirical studies that estimate the effects of temperature and temperature‐diet interactions on feeding behavior and life history will be particularly valuable here. For example, increased temperature and reduced diet quality may have contrasting effects on meal size, switching rates, hormone degradation, and rate of mass gain, resulting in complex changes in mean and variation in final size and development time (Legault & Kingsolver, 2020). Our model provides a quantitative and predictive framework for exploring these connections among feeding behavior, environmental conditions, and life history traits.

## AUTHOR CONTRIBUTIONS

**Anna L. Parker:** Conceptualization (equal); data curation (equal); formal analysis (equal); investigation (supporting); methodology (equal); project administration (lead); supervision (lead); visualization (lead); writing – original draft (lead); writing – review and editing (equal). **Anna Albright:** Conceptualization (supporting); data curation (equal); formal analysis (supporting); investigation (lead); methodology (supporting); visualization (supporting); writing – original draft (supporting); writing – review and editing (supporting). **Joel G. Kingsolver:** Funding acquisition (lead); methodology (supporting); project administration (supporting); resources (lead); supervision (equal); writing – review and editing (equal). **Geoffrey Legault:** Conceptualization (equal); formal analysis (lead); methodology (equal); supervision (supporting); validation (lead); visualization (equal); writing – review and editing (equal).

## CONFLICT OF INTEREST STATEMENT

The authors declare no conflict of interest.

## Supporting information


Appendix S1

## Data Availability

All code and data can be found here: https://doi.org/10.5061/dryad.hqbzkh1md.
