# Supplementary material for: Predicting age and mass at maturity from feeding behavior and diet in Manduca sexta: An empirical test of a life history model
Source: Ecol Evol. 2023 Feb 22;13(2):e9848. doi: 10.1002/ece3.9848 (PMC9944182; doi:10.1002/ece3.9848)
Supplement: Supplementary file 1 — Appendix S1 [file ECE3-13-e9848-s001.docx]

### **Appendix**


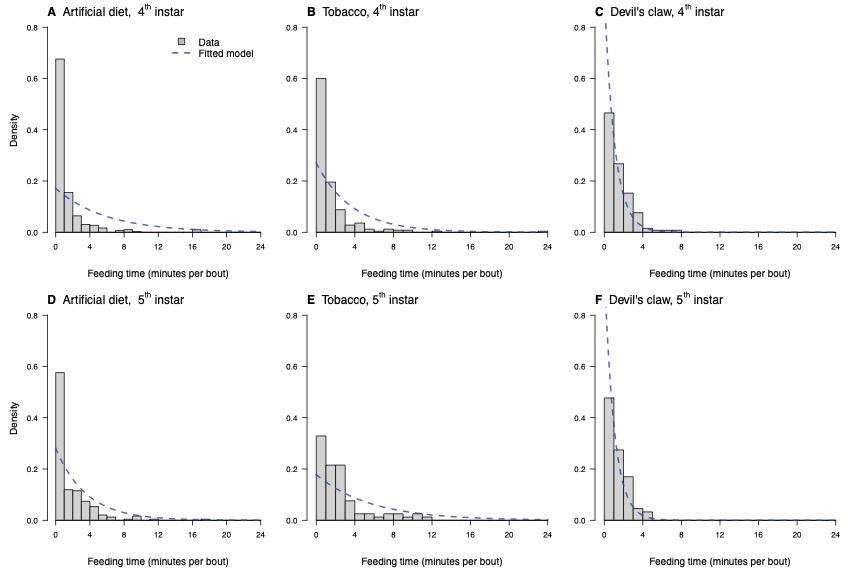


**Figure S1:** Histograms of the length of each feeding bout across instars and diet type (gray bars). The blue dashed lines show the distribution of feeding bout length from the fitted model.


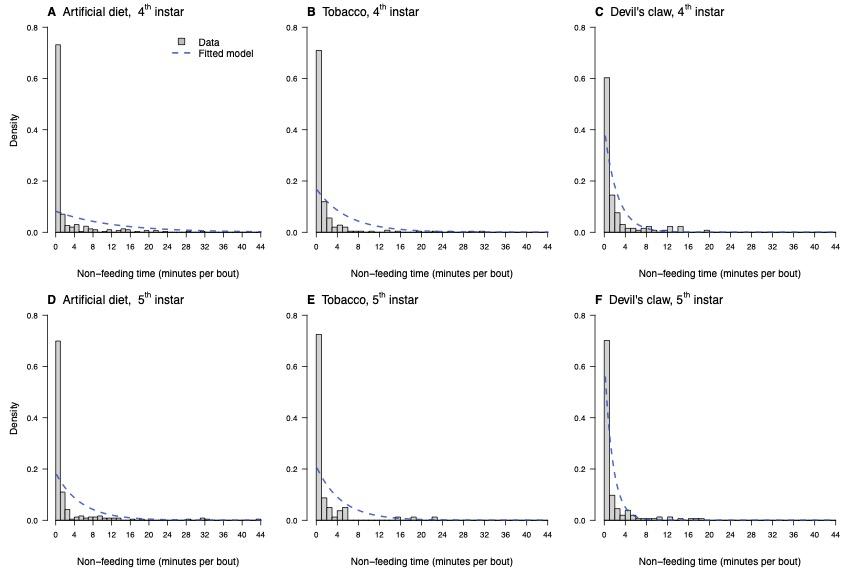


**Figure S2:** Histograms of the length of each non-feeding bout across instars and diet type (gray bars). The blue dashed show the distribution of non-feeding bout length from the fitted model.


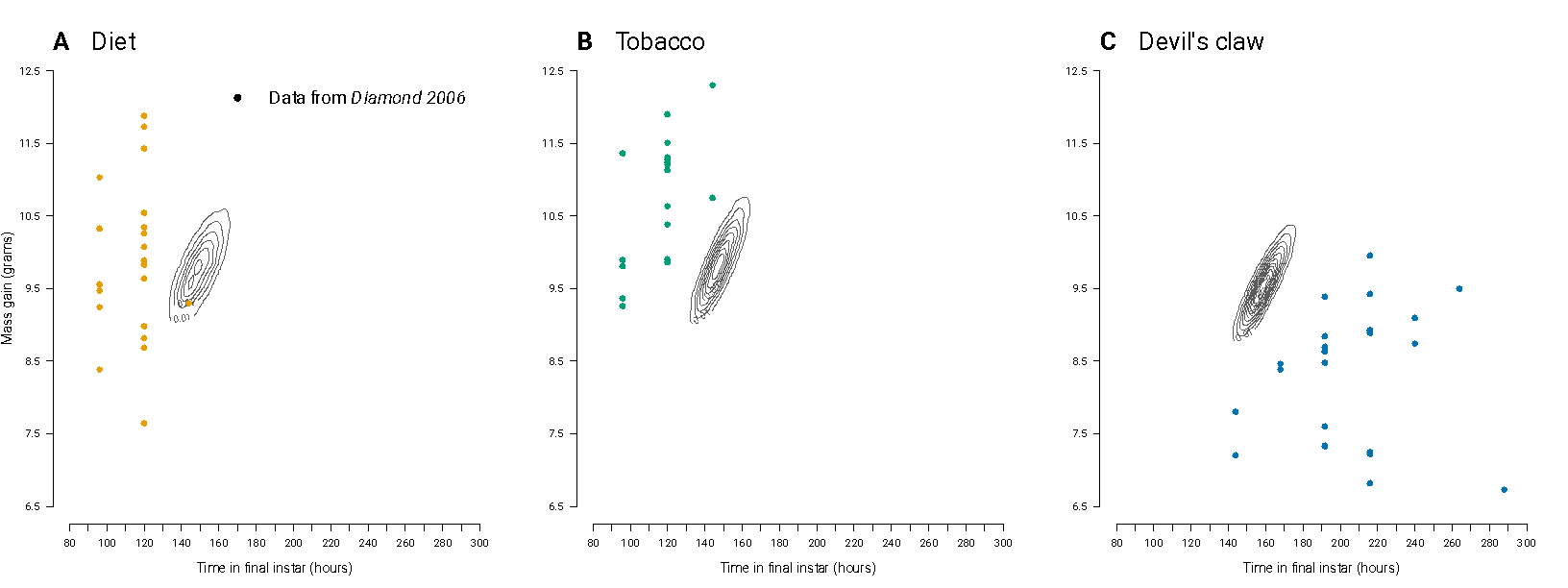


**Figure S3**: Plots comparing empirical data to model predictions when mass gain is fixed to the population-level mean. The black contours represent the distribution of masses and ages at maturity (wandering) predicted by the model for individuals raised on artificial diet (A), tobacco (B), and devil’s claw (C). The colored points denote empirical growth metrics collected from independent datasets from previously-published Kingsolver lab studies (Diamond & Kingsolver, 2010a; Kingsolver et al., 2009).
